# Fragmentation of Beaded Fibres in a Composite

**DOI:** 10.3390/ma15030890

**Published:** 2022-01-24

**Authors:** Carol Winnifred Rodricks, Israel Greenfeld, Bodo Fiedler, Hanoch Daniel Wagner

**Affiliations:** 1 Department of Materials and Interfaces, Weizmann Institute of Science, Rehovot 7610001, Israel; carol.rodricks@weizmann.ac.il (C.W.R.); israel.greenfeld@weizmann.ac.il (I.G.); 2 Institute of Polymers and Composites, Hamburg University of Technology, D-21073 Hamburg, Germany; fiedler@tuhh.de

**Keywords:** interface, beaded fibre composite, structural modification, mechanical interlocking, fragmentation test

## Abstract

The fibre–matrix interface plays an important role in the overall mechanical behaviour of a fibre-reinforced composite, but the classical approach to improving the interface through chemical sizing is bounded by the materials’ properties. By contrast, structural and/or geometrical modification of the interface may provide mechanical interlocking and have wider possibilities and benefits. Here we investigate the introduction of polymer beads along the interface of a fibre and validate their contribution by a single fibre fragmentation test. Using glass fibres and the same epoxy system for both matrix and beads, an increase of 17.5% is observed in the interfacial shear strength of the beaded fibres compared to fibres with no polymer beads. This increase should lead to a similar improvement in the strength and toughness of a beaded fibre composite when short fibres are used. The beads were also seen to stabilise the fragmentation process of a fibre by reducing the scatter in fragment density at a given strain. A case could also be made for a critical beads number—4 beads in our experimental system—to describe interfacial shear strength, analogous to a critical length used in fibre composites.

## 1. Introduction

The mechanical behaviour of fibre-reinforced composites is as dependent on the characteristics of its fibre–matrix interface as on the properties of its constituent materials [1,2,3,4]. Good fibre–matrix bonding at the interface, for instance, ensures efficient stress transfer in shear from the matrix to the fibre and results in strong, yet typically brittle, composites. Weak fibre–matrix bonding, in contrast, allows for redistribution of stresses around defects and cracks and for good energy dissipation, and results in tough yet weak composites. Tuning of the mechanical behaviour of a fibre-reinforced composite can be done through the modification of the interface. Usually, this modification is done chemically through polymer sizings or coupling agents applied to the surface of the fibre [4,5,6], but the degree to which mechanical behaviour tuning is possible is limited by the basic properties of the materials used. Furthermore, the strength and toughness achievable by such tuning are typically mutually exclusive of each other, and the enhancement of either property is usually accompanied by the degradation of the other [7].

A number of studies have emerged on the improvement of the mechanical behaviour of composites through the structural design of components [8,9,10,11,12,13,14,15,16,17]. Mechanical interlocking at the interface of these components is often cited as an effective means by which superior mechanical properties and an optimal balance of strength and toughness may be obtained [8,11,12,14]. This approach of property tuning through the structural design of components draws its inspiration from building strategies seen in natural composites. Composite materials in nature have a fairly limited selection of relatively weak component materials, yet due to the complex hierarchical architectures in which these component materials are put together, their overall mechanical properties far exceed the basic rule of mixtures [7,12,18,19,20]. Nacre [18,19], bone [12,21], bamboo [12], teeth [12] and feathers [22,23] are a few examples of natural composites where this trend is seen. The interfaces in natural composites significantly control deformation and fracture, though they only occupy a small volume fraction of the whole material [18].

Recently, we introduced a new interfacial structure of intermittently spaced polymer beads at the interface of fibre-reinforced composites [8,9,10]. Epoxy droplets are deposited along the surface of a glass fibre, resulting in a fibre that is no longer classically cylindrical, but rather has a wave-like topography. Single fibre pullout tests revealed that the epoxy beads work as topographical obstacles through mechanical interlocking, improving stress transfer through friction shear from the matrix to the fibre, and increasing resistance of the fibre to pullout, thus potentially improving strength and toughness simultaneously in a composite [8]. A natural analogy of such a structure is found in the rachis (shaft) of bird feathers, where periodically spaced nodes are observed on the fibres of the natural composite [22,23]. These nodes are thought to improve the transmission of forces to the fibres and resistance of the fibres to pullout [23], in similarity to the hypothetical mechanisms by which polymer beads at the fibre–matrix interface are thought to simultaneously improve the strength and toughness of a fibre-reinforced composite.

Our work on beaded fibres has been progressive. We first introduced the concept of beaded fibres and qualitatively analysed and predicted the effect of beads at the interface [10]. We then modelled and simulated the effect of beads at the interface assuming different materials for the bead and the matrix [9]. Our recent work quantitatively analysed the pullout behaviour of a single beaded fibre and put forth a phenomenological model to describe the mechanism by which the beads simultaneously increase strength and toughness in a model composite [8]. Here we continue our investigation into the mechanical interlocking effect of the beads, this time investigating the quantitative effect of multiple beads along the interface of a continuous fibre using the single fibre fragmentation test (SFFT) method [3,6,24,25,26,27,28,29]. SFFT of beaded fibres was already demonstrated in [10] but without quantitative measurements and evaluation of the beads’ effectiveness; the current study aims at filling this knowledge gap. SFFT is a common technique for analysing the interface of model composites owing to the fact that interfacial behaviour can be observed and data collected over a large area of the interface. The process is also thought to be similar (to a certain extent) to the in situ events in an actual composite [25]. As in the previous experimental studies on polymer beads at the interface [8,10], the beads and the matrix are made from the same epoxy system so that the neat effect of the geometry would be identified. Particular attention is paid to the effects of the beads on the effective interfacial shear strength and on the fragmentation behaviour of beaded fibres compared to regular fibres. The significance of the results towards a full-scale fibre-reinforced composite is discussed.

## 2. Materials and Methods

### 2.1. Materials

E-glass fibres (S139, Vetrotex, Saint-Gobain, Chambéry, France) with an average diameter of 16.8 µm were used in this study. The fibres were washed in acetone several times and dried in an oven for 1 h at 80 °C to remove surface impurities. The transparent epoxy system chosen for this study was EP828, a bisphenol-A diglycidyl ether with its compatible polyetheramine hardener, EPC304, both supplied by Polymer Gvulot Ltd., Gvulot, Israel. The resin and hardener were mixed in a weight ratio of 100:42. A centrifugal mixer with deaeration capabilities (Thinky ARE-250 CE., Thinky, Laguna Hills, CA, USA) was used to mix the epoxy mixture to ensure a homogenous mixture free of air bubbles. The beads and the matrix were both made from this epoxy system. The epoxy was cured for 6 h at 100 °C.

### 2.2. Beaded Fibre Preparation

**Figure 1:**
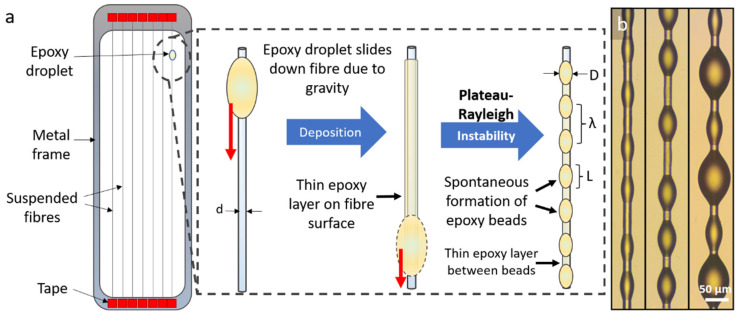
**Preparation of beaded fibres.** (**a**) Formation of fibre with intermittent epoxy beads through Plateau–Rayleigh instability [8,9,10]. A droplet of epoxy is placed on a suspended fibre of diameter d. As the droplet slides down the fibre, a thin layer of epoxy is formed, which spontaneously partitions into fairly uniformly spaced beads of length L, diameter D and wavelength λ. A thin layer of epoxy is observed between beads. (**b**) Cured EP828 epoxy beads of various diameters on E-glass fibres. Alternating large and small beads are typically seen when bead diameter > 70 µm.

Beaded fibres were prepared by taking advantage of the Plateau–Rayleigh instability [8,9,10]—a phenomenon by which a liquid cylindrical film spontaneously partitions into approximately evenly spaced droplets [10]. Taut glass fibres were glued to a metal frame using tape, as seen in Figure 1a. Using another small fragment of a glass fibre, a droplet of epoxy that was mixed and deaerated was deposited onto each suspended fibre. As each droplet slid down the fibre under gravity, a thin uniform layer of epoxy was deposited onto the surface of the fibre. Through the Plateau–Rayleigh instability, the layer then almost instantaneously and completely spontaneously separated into fairly evenly spaced, similarly sized beads along the entire length of the fibre. The epoxy beaded fibres were then moved to an oven and cured for 6 h at 100 °C. For further details on the process and the instability, refer to [10].

Beaded fibres obtained from this method can be seen in Figure 1b. The beads are fairly uniformly sized, and the bead parameters (diameter, length and wavelength) were successfully regulated by controlling the size, viscosity and surface tension of the initial epoxy droplet applied to the fibre, which in turn affected the thickness of the epoxy layer deposited on the fibre once the droplet had slid down it. A larger initial droplet resulted in a thicker epoxy coating being deposited on the surface of the fibre, which in turn resulted in larger beads with longer wavelengths. A more viscous drop would have had a similar effect. A smaller initial droplet resulted in a thin epoxy layer on the fibre and consequently smaller beads with shorter wavelengths. Typically for beads of diameter greater than 70 µm, smaller beads are seen to alternate between the larger beads. This is due to the fact that an epoxy layer is present on the surface of the fibre between beads [8,10], and for bigger bead diameters, this epoxy layer is sufficiently thick for a secondary instability to occur. For this study, fibres with a bead diameter range of 28–35 µm were chosen, with the average bead diameter taken as being approximately 32 µm. The corresponding bead length range was 60–70 µm, and the bead spacing (wavelength, bead-centre to bead-centre) was 90–160 µm. These bead parameters were chosen because they are more uniform in size.

An alternative method for controlling the bead parameters, which was used successfully in our previous studies [9], involved dipping and drawing out taut glass fibres from an epoxy bath at a controlled velocity. The thickness of the epoxy layer on the fibre (and thus the bead parameters) was determined by the resin viscosity and surface tension, and speed at which the fibres are drawn out of the epoxy bath. In this method, higher draw-out velocities or more viscous resins resulted in a thicker epoxy layer, which resulted in larger beads with higher wavelengths, and vice versa [9].

### 2.3. Single Fibre Fragmentation Tests

#### 2.3.1. Background

**Figure 2:**
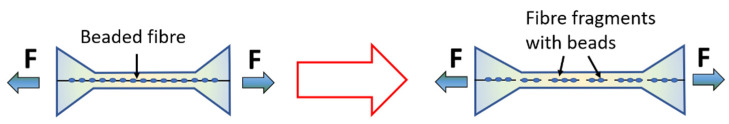
**Schematic representation of a single fibre fragmentation test for a beaded fibre.** Dog-bone shaped epoxy sample with embedded fibre is subjected to tension until fibre fragmentation occurs. Fibre fragments are hypothesised to have beads present on them.

In the single fibre fragmentation test method, a fibre is embedded in a polymer matrix that is usually shaped in the form of a dog-bone for ease of handling and testing (Figure 2). The specimen is placed in a tensile tester and elongated, and as the applied strain increases, the fibre breaks at points along its length where the cumulative shear stress induced at the interface exceeds the fibre strength. Applying further strain, the fragmentation process continues until a stage is reached where the lengths of the fragments are too short to allow a sufficient build-up of stress to equal or exceed the fibre tensile strength. At that stage, no more fragmentation occurs even with further elongation, and the process is said to have reached saturation [3,24,27].

An important parameter obtained from a typical single fibre fragmentation test is the critical length, $L_{c}$, which can be thought of as the maximum length a fibre fragment can have before the stress induced via the interface exceeds the fibre tensile strength and breaks it. At saturation, the length of fibre fragments is then anywhere between $\frac{1}{2}L_{c}$ and $L_{c}$. Therefore, the average length of fragments at saturation, $L_{sat}$, is equal to $\frac{3}{4}L_{c}$. The effective interfacial shear strength, τ, can be estimated by a simple force balance equation shown in Equation (1). This estimation is based on the constant shear model proposed by Cottrell, Kelly and Tyson [25,30,31].

(1) $$\tau =\frac{{\bar{\sigma }}_{\left(L_{c}\right)}r}{L_{c}}=\frac{3{\bar{\sigma }}_{\left(L_{c}\right)}r}{4L_{sat}}$$

where ${\bar{\sigma }}_{\left(L_{c}\right)}$ is the average strength of the fibre at critical length and r is the fibre radius. The critical length is unique to a particular fibre–matrix system, and changes in it can be used as a preliminary indication for the direction in which the interfacial characteristics have moved. Shorter critical lengths are typically associated with a fibre–matrix system with good interfacial adhesion, and vice versa. If, for instance, a coating is applied at the interface of a fibre–matrix system and an increase in critical length is observed, it is highly probable that the interfacial adhesion (that is, shear strength) of the system has been reduced, since they are inversely related, as seen in Equation (1). Typical critical lengths are less than 1 mm and therefore ${\bar{\sigma }}_{\left(L_{c}\right)}$ cannot be determined experimentally, but is calculated from the Weibull parameters obtained for the same fibres at higher gauge lengths. A full explanation of the procedure of calculating ${\bar{\sigma }}_{\left(L_{c}\right)}$ may be found in the Appendix A.

#### 2.3.2. Specimen Fabrication

A total of 24 samples with beaded fibres were made and tested in this study. The results were compared to 24 bead-less fibre samples (i.e., glass fibres with no beads), which functioned as the control for the test.

**Figure 3:**
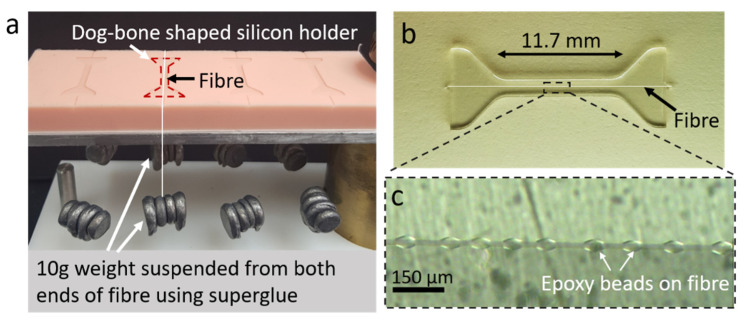
**Single fibre fragmentation test–sample preparation.** Fibre highlighted in white to show position. (**a**) Fibres are placed along the centre of a dog-bone shape mould and 10 g weights are suspended from both ends using super glue. Epoxy is then added and cured. (**b**) Dog-bone shaped sample after curing. Dimensions of the area tested are 11.7 × 1.0 × 2.0 mm. (**c**) Embedded beaded fibre in epoxy viewed under polarised light microscope, showing no observable voids.

Samples for the single fibre fragmentation test were made by placing a single fibre in the centre of a dog-bone shaped silicon mould and attaching 10 g weights to both ends of the fibre (Figure 3) using a quick-drying cyanoacrylate glue (CN, Tokyo Measuring Instruments lab, Tokyo, Japan). This particular cyanoacrylate glue was chosen because it could withstand the curing cycle and thus ensure that the weights stayed on the ends of the fibre throughout the cure cycle. Pre-mixed and deaerated epoxy was then added to the moulds to cover the fibre, and the moulds were shifted to an oven and cured for 6 h at 100 °C.

The pre-tensioning of fibres by hanging 10 g weights on their ends during sample fabrication is an important step to ensure fragmentation saturation is achieved. This is due to (1) glass fibres typically having a high strain to failure, and (2) the mismatch in thermal expansions of the epoxy and the fibre resulting in a compressive stress being applied to the fibre once the sample is cooled down to room temperatures after curing [24,27,29,32,33,34,35,36]. This mismatch results in more strain being needed to fraction the fibre. Insufficient pre-tensioning (i.e., with insufficient weights) results in the saturation not being achieved. See Appendix B for the determination of the strain induced in the fibre due to thermal mismatch and the pre-tensioning weights.

#### 2.3.3. Test Procedure

**Figure 4:**
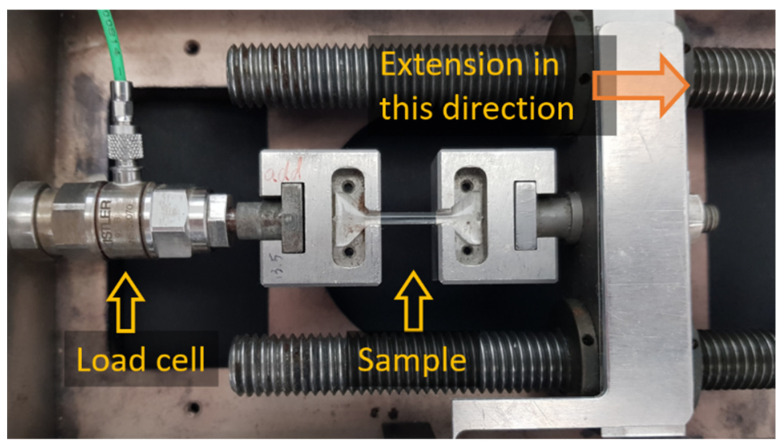
Set-up for single fibre fragmentation test.

The fragmentation tests were carried out on a Minimat tensile test instrument that was equipped with a 200 N load cell. The wide ends of the dog-bone epoxy sample were placed in a set of slots (Figure 4). Small weighted lids were placed over the wide ends of the sample in the slots so as to keep the sample in place during the test and especially after the matrix broke. The use of slots instead of clamps was so as to prevent the build-up of stress in the sample ends and to securely hold the sample in place without any slippage for the duration of the extension. Samples were extended until the sample broke, and the force–displacement curves of the samples were recorded. The displacement rate was 1 µm s^−1^. The number of breaks of a fibre in a sample was counted under an optical microscope only after matrix failure and sample rupture. A stereo-zoom fitted with a video camera and a cross-polariser was used to monitor the fragmentation process along the entire sample. The videos and the force-displacement curves for the samples were synchronised to correlate fragment density to percentage strain in the samples.

## 3. Results

### 3.1. Qualitative Analysis under Cross-Polarised Light

Embedded beaded fibres under load were qualitatively analysed under cross-polarised light. Transparent epoxy matrices are typically optically isotropic, but in highly stressed regions, such as those around fibre breaks, the matrix becomes optically anisotropic or birefringent and appears brightly lit and colourful compared to the low-stressed regions. Birefringence in epoxy matrices makes it possible to qualitatively study stress distributions at the interface of fibre–matrix systems.

**Figure 5:**
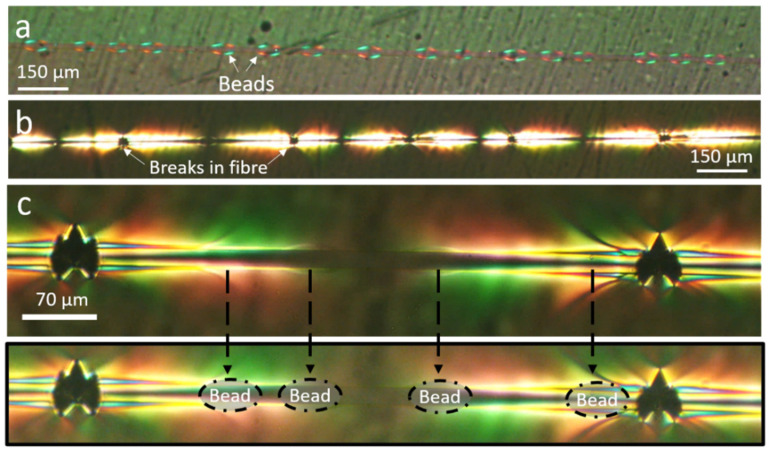
**Fragmentation behaviour of beaded fibres under load under cross-polarised light.** (**a**) Beaded fibre embedded in epoxy under load. (**b**) Birefringence pattern of a beaded fibre after fragmentation saturation. The black dots indicate fibre breaks. (**c**) Beaded fibre fragment between fibre breaks with bead positions outlined in dotted lines below.

Figure 5a presents a beaded fibre under load before any fibre fragmentation has occurred. Some birefringence is observed at the apparent bead–matrix interface, implying that there is some stress discontinuity (that is, high stress gradient) across this interface under load.

The fact that there is a distinct interface between the bead and the matrix also implies that they behave as two separate entities rather than as one continuous phase, despite being made from the same epoxy system. If the beads and matrix had constituted a continuous phase, the stress at the interface would not be discontinuous but instead continuous as in bead-less fibres, without the ‘butterfly’ pattern. This is in agreement with our previous observations on the pullout of beaded fibres [8,10].

Figure 5b shows a beaded fibre after several fibre breaks at saturation (see definition in Section 2.3.1). Saturation at this point was presumed because (1) no more fibre breaks were observed at this stage and (2) the ends of the birefringent patterns were seen to almost touch each other, which is an indication of saturation of the fragmentation process being reached [24]. Figure 5c displays a fragment of a beaded fibre between two fibre breaks while still under load. The positions of the beads are marked below for more clarity. No debonding is observed between the bead and the matrix (debonding typically appears as a black shadow). It is interesting to note that all the beads are also brightly lit, particularly the two beads far from the centre of the fragment. The pattern of lighting in the bead appears to be distinctly brighter than the matrix surrounding it, implying that there is some stress concentration in the bead compared to the matrix in that region. Such stress concentration indicates that the bead is bearing high stress, acting as an obstacle against matrix displacement, thereby mechanically interlocking the fibre. A video of the fragmentation process of beaded fibres has also been included in the Supplementary Materials for further clarity. The video demonstrates the gradual rise in the beads stress level with respect to the surrounding matrix as the external load is increased.

**Figure 6:**
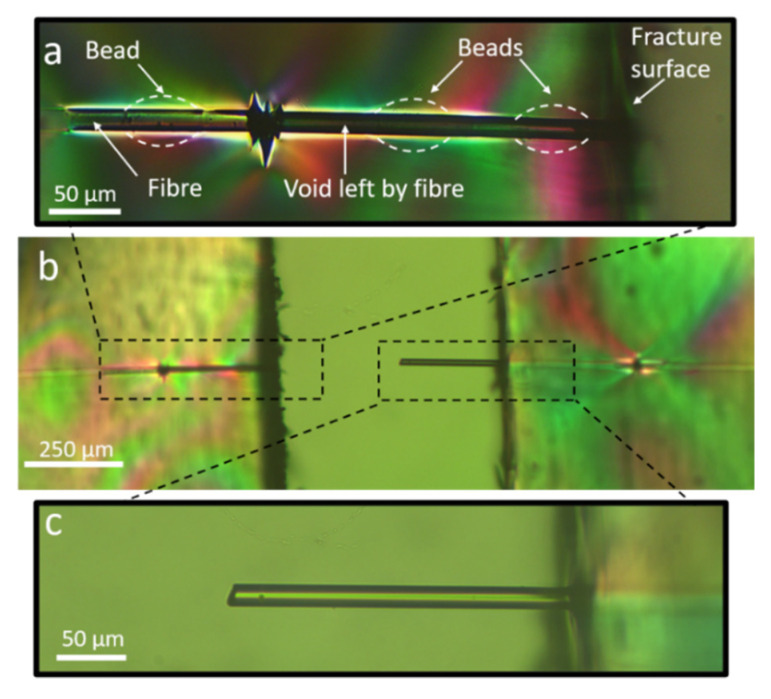
**Fibre pullout from single fibre fragmentation test of beaded fibres.** The beads are marked out with white dotted lines. The beads stay in the matrix (**a**) and are not pulled out with the fibre (**c**). The fibre and matrix are seen side-by-side in (**b**).

Some of the beaded fibre samples showed fibre pullout on completion of the test when the sample ruptured, as seen in Figure 6. The matrix close to the fracture surfaces was necked and thus birefringent, making the contrast between the beads and the matrix in this region weaker than in other parts of the sample. Therefore, for better clarity, the beads are marked out in white (Figure 6a). The fracture surface was seen to be perpendicular to the fibre (Figure 6b). The beads were not pulled out with the fibres but remained in the matrix. No epoxy residue was observed on the pulled-out fibre surface (Figure 6c), indicating that the failure between the bead and the fibre was adhesive and that between the bead–matrix and bead–fibre interfaces, the bead–fibre interface is likely the weaker interface. This particular observation was consistent with our previous work on the pullout of beaded fibres [8].

### 3.2. Fragmentation Behaviour and Effective Interfacial Strength

**Figure 7:**
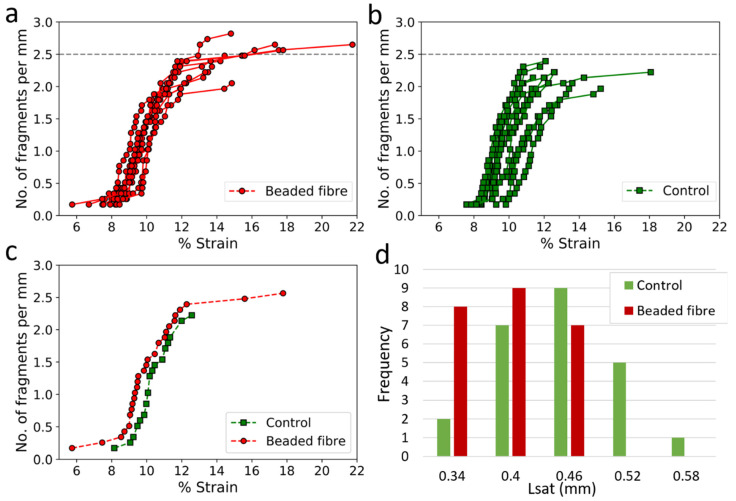
**Fragments density vs.% strain for** (**a**) Beaded fibres. (**b**) Bead-less fibres. (**c**) A comparison between typical behaviour of beaded fibre to bead-less fibre. The connecting lines are to identify each test separately. The error in strain is negligible. (**d**) Distribution of the fragment saturation length $L_{sat}$ for beaded fibres compared to the control.

Using a cross-polarised optical stereo-zoom, videos of the fragmentation process of randomly chosen 9 beaded fibre samples and 11 control samples were recorded to synchronise fibre breaks with the applied strain in the model composite sample. This was done to examine whether the beads influenced the fragmentation process of the fibre while under load. The number of breaks in each sample at a given strain was translated to fragments density and plotted against percentage strain in the composite (Figure 7a,b). No fitting was done for the data points in these graphs. For ease of comparison between the two configurations, Figure 7c presents a beaded fibre sample and a control, each showing typical behaviours for their batch. It was not possible to plot all the fibre breaks up to saturation. A very low magnification stereo-zoom was used so that the entire length of each specimen could be viewed. Fibre breaks were therefore identified by the birefringence pattern formed in the matrix around the break rather than by the break itself since the breaks were often too small to detect. Closer to saturation, fibre breaks were very likely formed close to other pre-existing breaks, and any birefringence produced by a new break was, in all likelihood, masked by the patterns already in the matrix. Nonetheless, fibre breaks during the beginning and middle of the fragmentation process could be seen clearly and thus studied. Hence, the plots of fragment density vs. strain provide good insights into how the beads affected the fragmentation process of fibres.

In Figure 7a,b, no fibre breaks were observed at strains below 5.5% in either configuration. However, some of the beaded fibre samples appeared to start fragmentation at lower strains (Figure 7a) than the control (Figure 7b), an expected outcome of the higher stress induced by the beads on the fibre. Fragmentation for the control typically began between 7.6 and 9.8% strain, whereas, for beaded fibre samples, fragmentation was seen to begin anywhere between 5.7 and 8.3% matrix strain. The beads appeared to ‘stabilise’ the fragmentation process since less scatter was observed among the beaded fibre samples compared to the control, meaning that the probability of failure at a given load can be predicted more accurately. The beaded fibre samples attained higher overall fibre fragmentation densities than the control, so that the fragment lengths for beaded fibres were shorter than those of the control, implying that stress transfer from the matrix to the fibre was better for the beaded fibre samples than the control. The curves, therefore, indicate a successful modification of the interface of a fibre in a matrix due to the presence of the beads.

The average number of breaks in the fibre at saturation was recorded for all 24 beaded fibre samples and 24 control samples after each of the samples had failed. The average fragment length at saturation, $L_{sat}$, was calculated for each sample by dividing the length of the fibre under fragmentation (11.7 mm; Figure 3) by the total number of breaks along the fibre of that particular sample. The average of each configuration is recorded in Table 1. Beaded fibres, on average, had a higher number of fibre breaks compared to the control and thus lower values of $L_{sat}$. Though the difference between the number of breaks in the fibre appears small, a *t*-test shows that it is still highly significant. A plot of the distribution of average $L_{sat}$ for beaded fibres compared to the control can be found in Figure 7d. While there is some overlap between the samples, it can be seen that the beaded fibre samples (red) tend to have lower $L_{sat}$ than the control. This concurs well with what was observed in the plots of fragmentation density vs. strain in Figure 7a,b, where higher fragment densities were observed for beaded fibre samples compared to the control.

$L_{c}$, the critical length and τ, the effective interfacial shear strength, were calculated by Equation (1) and also recorded in Table 1. The calculation of the fibre strength, ${\bar{\sigma }}_{\left(L_{c}\right)}$, may be found in Appendix A. ${\bar{\sigma }}_{\left(L_{c}\right)}$ was calculated from the Weibull parameters of fibres without beads since the beads and the matrix were made from the same material. From our previous study [8], the bead and the matrix were found to have identical physical and chemical properties, and thus, the system can ultimately be considered as a single fibre embedded in bulk epoxy. The strength of the fibre embedded in the matrix would therefore not be affected by the presence of the beads. The effective interfacial shear strength for beaded fibre was likewise calculated by taking only the fibre radius into consideration and not the radius of the beads since the beads and the matrix were made from the same epoxy material. Therefore, calculations of τ were done only taking into account the fibre radius for both the control as well as beaded fibres.

A highly significant increase of 17.5% was calculated in the effective interfacial shear strength of beaded fibres compared to bead-less fibres. The beads, therefore, improved the effective interfacial adhesion of a fibre in a matrix. It must be noted that the calculations of τ for beaded fibres and the control were done using a constant shear model (Equation (1)), which is a simplistic model, and therefore the term ‘effective’ is used to describe the interfacial strength. We may expect a comparable improvement in strength and toughness (pullout energy) in composites reinforced by beaded fibres when short fibres are used (shorter than the critical length), as both properties are proportional to the interfacial strength [37]. When longer fibres are used, the strength will still improve, whereas the pullout energy might degrade; however, such degradation should be outweighed by the dissipation of plastic and friction energy at the bead–matrix interface due to relative motion between them (see more on this in the discussion, in Section 4.1).

### 3.3. Distribution of Breaks and Critical Number of Beads

**Figure 8:**
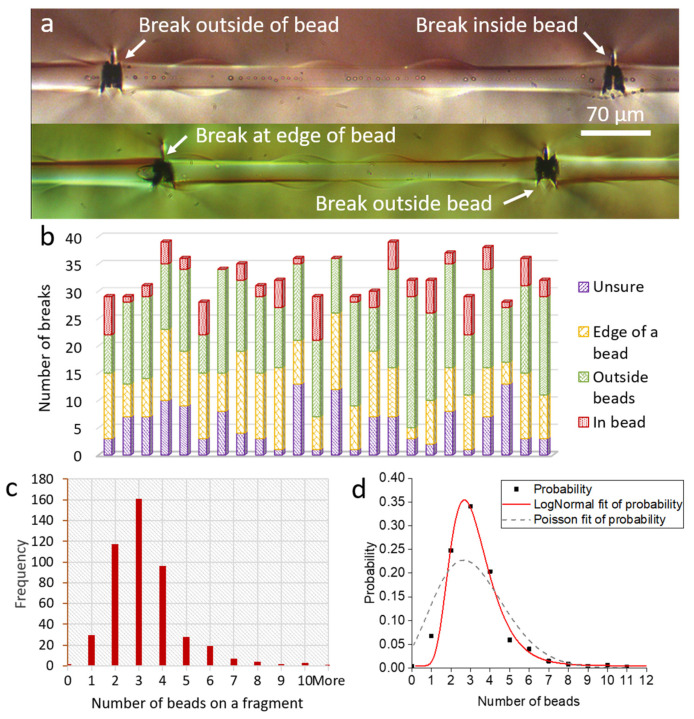
**Analysis of beaded fibre fragments at saturation**. (**a**) Beaded fibres after fragmentation under polarised light showing fragments with three whole beads (top) and four whole beads (bottom). Breaks are seen in between beads, at the edge of a bead and inside a bead after fragmentation test. (**b**) Position of breaks in beaded fibre samples. (**c**) Number of beads on each fragment. (**d**) Lognormal fit to data from (**c**), with mean of 3.05 and standard deviation of 0.36.

To further probe the role of the beads at the interface, the distribution of the position of breaks along a beaded fibre for all the beaded fibre samples was studied. Both distributions are displayed in Figure 8. Figure 8a presents fragments of beaded fibres. The fragment on top is between a break outside a bead (left) and a break inside a bead (right), and the fragment below is between a break at the edge of a bead (left) and a break outside a bead (right). Three whole beads are seen on the fragment on the top, and four whole beads are seen on the fragment below. Figure 8b provides a distribution of the position of fibre breaks for each of the beaded fibre samples. Most of the fibre breaks (44% of all fibre breaks) were found to be outside and far away from beads (green), whereas a large fraction of breaks (29%) was found at the edge of beads (yellow). Only a few breaks were found to be completely inside beads (10%). The matrix cracks caused by fibre breaks were occasionally larger than the beads (as seen in Figure 5c), making it impossible to determine whether a fibre break was in the bead, outside of it or at its edge. These breaks were labelled as ‘unsure’ on the graph (blue) and accounted for 17% of the fibre breaks in beaded fibres. So, excluding the uncertain cases, 53% of breaks were outside beads, whereas 47% were either inside beads or at their edge; given that the fibre length covered by beads is on average the same as the length not covered by beads, and that the beads distribution along the fibre is widely varied (see Section 2.2), this implies that the locations of breaks and beads are not correlated.

The distribution of the number of beads on fibre fragments was also studied. Figure 8c shows the total number of beads on the fragments of 15 beaded fibre samples after the completion of the fragmentation test. We observe that the number of beads on a fragment at saturation is not random, but appears to follow a log-normal distribution (Figure 8d). The most frequent number of beads on a fibre fragment was $n_{sat}=$ 3 beads, followed by 2 beads and 4 beads per fragment. In a previous study [10], we had put forth a concept of critical number of beads, $n_{c}$, where the maximum stress in the fibre is determined by the number of beads on the fragment rather than by the length of the fibre. The critical number of beads would be a discrete quantity that could replace the critical length $L_{c}$. For this particular system, the critical number of beads is possibly $n_{c}=\frac{4}{3}n_{sat}$ = 4. The fact that this is an even number is in line with the observation that most fibre breaks are outside beads (had the critical number of beads been odd, further breaks would most likely have occurred in the centre of the middle bead due to symmetry).

## 4. Discussion

### 4.1. Mechanical Interlocking

An increase of 17.5% in the effective interfacial strength, τ, of beaded fibre samples compared to control is quite a surprising outcome since the beads and the matrix are made from the same epoxy system. Supposedly, the beads and the matrix should have acted as one entity, and there should not have been a difference in the fragmentation behaviour or interfacial shear strength of the beaded fibre samples compared to the control. However, this is not what we observe. Instead, we not only see a very significant increase in τ of beaded fibres compared to the control, but we also see that the number of beads on fragments is not random.

The first possible hint as to why we observe these results is seen in Figure 5a, where we see a strong indication that the beads and the matrix act as two separate entities under load, with a distinct interface between them. From our previous work on the pullout of beaded fibres [8], we know that the epoxy beads are fully cured before they are embedded in the matrix during the sample fabrication process in Section 2.3.2. It is, therefore, less likely for the epoxy in the bead to form crosslinks with the epoxy in the matrix during sample fabrication because diffusion of epoxy oligomers from the liquid matrix into the solid bead is very limited, and most bonding sites at the bead side are already occupied, culminating in the interface between the bead at the matrix. This relatively weak interface is reflected in the lower slope of the force–displacement curve (at high strains) during pullout of a beaded fibre compared to a bead-less fibre. It must be noted that though the beads undergo the curing cycle twice first when the beads are formed on the surface of a fibre, and again when the beads are embedded in the matrix and cured—we know from our previous study on the pullout behaviour of beaded fibres, that the mechanical and chemical properties of the beads are identical to those of the matrix [8].

**Figure 9:**
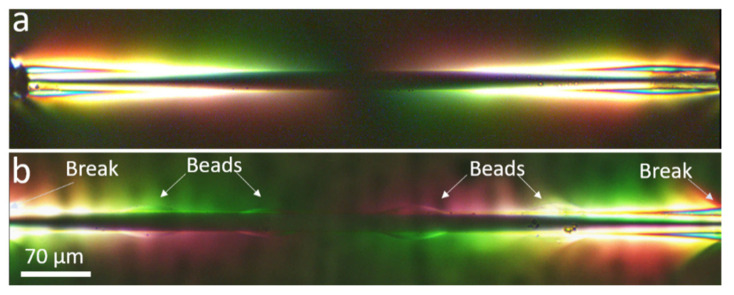
**Fibre fragments between fibre breaks before saturation.** (**a**) Control. (**b**) Beaded fibre.

A second hint is realised by comparing a fragment of beaded fibre between breaks and a bead-less fibre, as in Figure 9. Here, the beads are brightly lit, particularly closer to fibre breaks (which are just outside the frame of the images). This indicates that the beads are under higher stress with respect to the surrounding matrix when under load, implying that the beads act as mechanical interlocks for the fibre against the displacement of the matrix.

We previously put forth a phenomenological model based on mechanical ‘friction lock’ to describe the behaviour of beaded fibres under load [8]. Since the bead is fully cured and is not likely to form a significant number of bonds with the matrix, some relative motion is possible at the bead matrix interface. Thus, when a beaded fibre is subject to a load, it is thought to shift and push against the matrix, resulting in an equal and opposite stress being exerted on the bead by the matrix. This stress is converted to radial pressure in the bead, which then propagates through the bead to the bead–fibre interface, inducing a friction shear stress ($\tau_{f}$) at the bead–fibre interface. This friction shear stress is in addition to the existing bonding shear stress between the bead and the fibre, $\tau_{i}$, and so contributes to increasing the total effective τ of the system. The mechanism can be thought of as being very similar to the friction locking of mechanical parts using a wedge. Since the mechanism proposes a friction shear stress, which adds onto the bonding shear stress, the use of total effective shear stress τ to quantify the effect of beads at the interface in Section 3.2 is valid. This train of causes and effects is supported by the two experimental observations described above, namely the existence of a distinct interface, which is most likely weaker than the continuous epoxy, and the stress concentration in the beads with respect to their matrix surrounding. A detailed description of the phenomenological friction lock mechanism may be found in [8].

**Figure 10:**
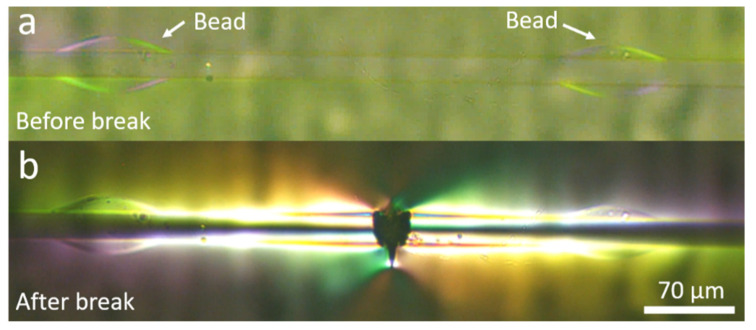
**Beaded fibre fragment under load** (**a**) before and (**b**) after fibre break. Before fibre break, no residual stress is observed inside beads. After fibre break, beads show stress concentration.

The mechanical locking action induced by the beads is demonstrated in Figure 10, which shows a portion of a beaded fibre before and after a fibre break. Before fibre break the stress level in the beads is moderate because the load is distributed over several beads along the fibre. However, after fibre break the stress at the two beads at both sides of the break, which are closest to the edges of the two new fibre fragments, rises distinctively over the matrix stress, reflected by the high light intensity in the beads. The higher stress in the beads with respect to the matrix implies that they are incurring extra load, indicative of their mechanical locking action. A similar observation is seen in the video of beaded fibres, which is found in the Supplementary Materials.

A question arises as to whether the effects we see in Section 3.2, namely the increase in fibre breaks (and consequently increase in interfacial shear strength) in beaded fibres, are because of stress concentrations inside the bead due to double curing of the bead and/or thermal mismatch, or due to the friction lock mechanism we briefly described here. The beads undergo a second cure cycle during sample preparation for the single fibre fragmentation test, and it is known that residual strains are possible in the fibre due to a mismatch in thermal coefficients of expansion of epoxy and glass fibres.

To answer this question, we first consider the effect of double cure on the bead properties. We refer back to our work on the pullout of single beaded fibres in [8], where we studied in detail the effect of a single bead at the fibre–matrix interface (we also refer to ref [8] for characterisation tests of the epoxy system used here). In ref [8], we addressed the issue with the bead undergoing a cure cycle twice and concluded that the properties of the epoxy in the bead did not significantly differ from that of the matrix. While tests on bulk epoxy that had been cured twice showed a slight increase in its glass transition temperature, $T_{g}$, compared to epoxy that had been cured once, there was no significant difference between the mechanical properties of epoxy that had gone through the cure cycle twice and that which had gone through the cycle only once. Bulk epoxy that was cured twice (so as to emulate the cure conditions of the bead) was found to have a slight (2.5%) but insignificant (*p*-value = 0.43) increase in tensile modulus. Running this increase in bead modulus in our micro-mechanical model described in ref [9], by setting the bead material stiffness higher by 2.5% than the matrix, an increase in effective interfacial shear strength of less than 0.1% was obtained. Such increase is negligible and nowhere near the very significant (*p*-value = 0.002) increase of 17.5% in the effective interfacial shear strength observed in Section 3.2. Moreover, referring to our work in [8], we observed an increase in pullout force and work with decreasing bead size (down to a limit that was not reached in these tests) because smaller beads enhance the wedging effect of the friction lock mechanism. Had the increase in pullout work and force simply been an artefact of stress concentration due to the bead being cured twice, bigger beads would have resulted in higher pullout forces and work. However, this was not observed.

Next, with regard to stress concentrations in the bead due to residual thermal stresses, we refer to our calculations in Appendix B, where the residual strain induced in the bead due to double curing, and the residual strain induced in the fibre due to the bead, were calculated and found insignificant. Thus, referring to the measures taken to relieve thermal strains during matrix curing (Section 2.3.2), and to our calculations in Appendix B, the 10 g weights used to offset thermal residual strain in bead-less fibres would be sufficient to offset any thermal residual strain induced in the fibre by the beads and the matrix as well.

We also refer to Figure 3c and to our video in the Supplementary Materials, where we see beaded fibres embedded in epoxy under polarised light but not under load. In these images, the birefringent pattern is not strong and appears mainly on the bead surface, indicating a distinct interface between the beads and the matrix. It is only once the beads are under load that a significant birefringence is observed. No obvious stress concentration is seen inside the bead before a fibre break, as seen in Figure 10. If the increase in effective interfacial shear stress was simply due to an artefact of residual stress in the bead, we would expect to see significant birefringence in the bead before a fibre break. Furthermore, Figure 5 and Figure 9b show fragments of beaded fibres between fibre breaks, and it can clearly be seen that the distribution of the stress concentration in the beads is not uniform in all beads. The beads on the outer edges (closest to fibre breaks) have higher stress concentrations, as reflected by them being more brightly lit, while the beads in the centre are not as brightly lit. Therefore, the hypothesis that the double cure of the epoxy or thermal residual stress in the bead is the reason for the results seen in Section 3.2 and Section 3.3 must be rejected.

A further question may arise as to whether the stress concentrations in the beads under load, whatever their source, induce local stress concentrations in the fibre, causing it to prematurely break and consequently disrupt the calculation of the effective shear strength in Equation (1). However, this hypothesis is not supported by the breaking statistics in Figure 8b, which shows that fibre breaks appear fairly randomly with respect to the location of beads. A stress concentration induced by the bead on the fibre would tend to occur consistently close to the bead edges, where the bead stress is maximal. Referring, for example, to Figure 9b and Figure 10, most fibre breaks are far from the bead edges. Furthermore, such hypothetical stress concentrations should be negligible compared to the stress build-up along the fibre length due to the cumulative effect of the shear stress applied by the matrix and the beads.

Yet, the most convincing evidence for the effectiveness of the beads in transferring stress from the matrix to the fibre, so that the stresses we observed are not a mere artefact of stress concentration, comes from our pullout tests in [8]. In these tests, which were carried out on a fibre fragment with a single bead, we measured an average increase of 0.15 N in the pullout force due solely to the bead, equivalent to an increase of about 30% in the effective interfacial shear strength. Hence, the possibility of an artefact should be rejected because an artefact would have decreased the pullout force rather than increase it. Furthermore, the following calculation accurately predicts the expected effective shear strength in the fragmentation tests, based on the results of the pullout test. Comparison of the effective interfacial strength of beaded fibres, τ, calculated from the pullout test in [8] and the fragmentation test presented herein, reflects a significant difference: the former predicts an increase of about 30% in τ compared to bead-less fibres (see Appendix C), whereas the latter predicts a mere 17.5%. We note that the pullout test was a pure test with only a single bead on a single fibre, compared to multiple beads in the fragmentation test. The difference in τ becomes clear when observing the stress intensity profile along a fibre fragment, seen in Figure 9. The stress in both the beaded and bead-less cases is very high at the fragment ends (high light intensity) and gradually decreases toward the fragment centre, reminiscent of the well-known shear-lag stress distribution. The predicted contribution to the shear stress by a single bead is added on top of the shear-lag stress at the region of the bead so that the contribution of the outer beads is significant, whereas that of the inner beads is much lower. In other words, most of the 17.5% increase predicted by the fragmentation test is due to the two outer beads, out of the total of four beads on the fragment, resulting in a rough average contribution of about 15% (i.e., 30% × 2/4), whereas the additional 2.5% (i.e., 17.5–15%) are due to the inner beads.

### 4.2. Effect of Beads Size and Fibre Volume Fraction

In this study, we examined only a single size of beaded fibres, i.e., fibres with beads that ranged from 28–35 μm (approx. 32 µm on average) with similar bead densities. To further understand the role of the beads at the interface, other bead diameters and spatial distributions will also be studied in future work to see if there is an optimal bead size and distribution for any potential enhancement of the interfacial shear strength, and to understand the influence on the position of breaks or the critical number of beads on fibre fragments. The combination of these effects—bead size and beads distribution—is quite complex. Although, intuitively, a larger bead should provide stronger mechanical interlock, we have shown in our previous work [8] that when the bead diameter is much reduced, its wedging effect is more pronounced. Similarly, although it seems that a higher density of beads (smaller wavelength) should provide better mechanical interlocking, this is not necessarily the case, because as shown above, not all the beads are contributing equally to the overall interlocking.

The strength and toughness of a composite are linearly dependent on fibre volume fraction (V_f_). Maximum volume fraction is achieved when the fibres are tightly packed, often in the form of prepregs. Therefore, given the presence of the beads on a fibre, can a practical volume fraction be achieved for a beaded fibre composite? Typical continuous glass-fibre composites have volume fractions within the range of 30–70%, depending on the application. For instance, applications in aerospace using prepregs have volume fractions of about 60%, whereas applications using the hand-layup method, such as what is seen in boat building, would only have a volume fraction of 30–40%. From our previous study on beaded fibres [10], the maximum bead locking effect is achieved when the bead diameter is about 1.5 times the fibre diameter. At that size, according to the volume fraction analysis in [10], the achievable fibre volume fraction is 40–50%, depending on whether the packing is continuous (bead-to-bead contact) or staggered (bead-to-fibre contact), which is suitable for a variety of composite applications. A higher volume fraction is still achievable for beads with smaller diameters, provided that the expected reduction in locking effectiveness would be compensated by using beads made of a stiffer and stronger material than the matrix. With such beads, a staggered tight packing of beaded fibres, where each bead is in contact with a neighbouring fibre, could result in enhanced mechanical locking due to dovetailing (like that seen in nacre) when the material is under load [10]. We note that although the diameter of the beads used in this study was on average 2.2 times the fibre diameter (i.e., above the optimal size), its locking effectiveness was measurable and significant.

For short fibre (or discontinuous fibre) composites, the benefits of using beaded fibres may be more pronounced. First, for many applications with short fibres, such as sheet moulding composites for structural parts in the automotive industry, the fibre volume fractions are much lower than that of composites using continuous fibres and can be even as low as 22% [38]. Therefore, obtaining beaded fibre composites of practical volume fraction is feasible and is not impeded by the presence of beads on the fibre. Moreover, as seen in this study, not all the beads in the composite appear to bear stress to the same degree, such that the beads on the ends of short fibres are expected to bear a higher stress concentration. Thus, if short fibres are used, this would result in more ‘active’ beads, or more beads contributing to stress transfer to the fibre. Thus, beaded short fibres potentially improve the strength of the composite compared to using fibres with no beads. In fact, a similar structure has been studied in the past of fibres with enlarged ends known as bone-shaped short fibres [16], where strength increases were observed and attributed to better stress transfer through mechanical interlocking of the enlarged ends. Therefore, short-beaded fibre composites have ample potential, and as such, studies on such composites are anticipated in the future.

## 5. Conclusions

Polymer beads at the interface appear to be a promising way of increasing the interfacial shear strength of a fibre-reinforced composite. Using a single fibre fragmentation test in a model of glass fibres with epoxy beads embedded in epoxy matrix, an increase of 17.5% was observed in the interfacial shear strength of beaded fibres compared to the control. A similar improvement is expected for a multifibre composite. The beads, therefore, improved the effective interfacial adhesion of a fibre in a matrix. The beads also seem to make the fragmentation process more uniform and predictable. It was also seen that for beaded fibres, fragmentation started earlier. Most fibre breaks lay outside the beads, with many lying at the edges of beads, implying that the beads acted as stress concentrators. The beads and the matrix had a distinct interface between them, implying that despite being of the same material, they were not a continuous phase but functioned as two separate entities. Where fibre pullout occurred, the beads were not pulled out with the fibre but stayed inside the matrix. These findings suggest that the beads serve as interfacial obstacles against matrix displacement, providing a mechanical interlock for the fibres.

In this study, we limited ourselves to using epoxy beads that were chemically and physically identical to the matrix. This was done so as to isolate and investigate the effect of geometry alone. However, as predicted in our previous theoretical study [9], using beads of different materials could be beneficial and is something that should be studied and pursued. For instance, using a stiffer material for the beads could result in an overall increase in stiffness of the composite, and using thermoplastic materials for the bead could potentially increase the toughness of the overall composite. Future work anticipates a detailed model of how the beaded fibres behave under fragmentation, and a study into the effect of beads diameter and distribution on interfacial shear strength, critical bead number and fragmentation process of beaded fibres. Further tests are anticipated in this direction, including expansion to full-scale composites.

## Figures and Tables

**Table 1:** Effective interfacial shear strength.

	Control	Beaded Fibre	*p*-Value (*t*-Test)
Average # breaks at saturation	28.5 ± 3.6	32.8 ± 3.5	0.0002
Median # breaks	28.5	32	
$\mathrm{Saturation}\;\mathrm{length},\;L_{sat}$ (mm)	0.40	0.35	
$\mathrm{Critical}\;\mathrm{length},\;L_{c}$ (mm)	0.54	0.47	
$\mathrm{Fibre}\;\mathrm{strength},\;{\bar{\sigma }}_{\left(L_{c}\right)}$ (MPa) *	3179 ± 85	3272 ± 74	
Interfacial strength, τ (MPa)	50.7 ± 7.7	59.6 ± 7.8	0.0002

* Calculated in Appendix A.

**Figure A1:**
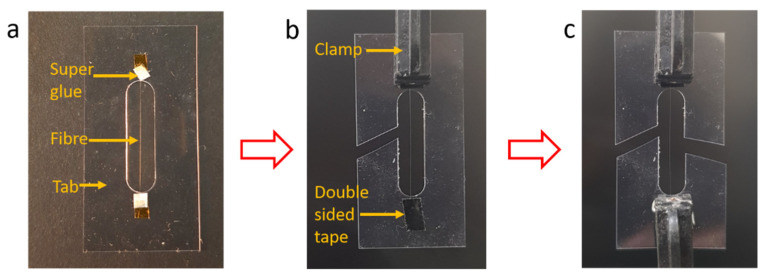
**Single fibre test sample preparation and testing**. (**a**) Fibre glued to plastic tab with super glue. (**b**) Double-sided tape is applied on top of glue and one side of tab cut before loading into clamps of the Instron. (**c**) Second side of tab cut so that fibre is free standing.

**Figure A2:**
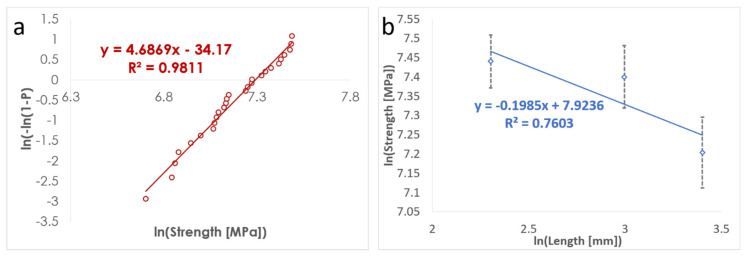
**Determining Weibull parameters using**: (**a**) Method 1: bead-less glass fibres in air at gauge length 30 mm. The slope of the linear curve denotes the Weibull shape parameter, m. The scale parameter, $\sigma_{0}$, is calculated by $e^{\frac{y\;intercept}{m}}$. (**b**) Method 2: bead-less fibres from gauge lengths 10, 20 and 30 mm. The slope of the linear curve is −1/m.

## Data Availability

Not applicable.

## Supplementary Materials

The following are available online at https://www.mdpi.com/article/10.3390/ma15030890/s1, Video S1: Single fibre fragmentation test: Beaded glass fibre embedded in epoxy.

## Appendix A. Determination of Average Fibre Strength at $L_{c}$ (${\bar{\sigma }}_{\left(L_{c}\right)}$)

### Background

The determination of the average fibre strength at critical fibre length, ${\bar{\sigma }}_{\left(L_{c}\right)}$, is a controversial subject [25]. The strength of reinforcement fibres is stochastic in nature and cannot be described by a single value as it depends on the sporadic presence of harmful defects. Therefore, the failure of the fibres is described by the weakest link model, which takes into account the fibre length and the density of critical defects. Reinforcement fibres, such as the E-glass fibres used in the current study, exhibit a size effect in which shorter fibres have higher tensile strengths due to the fact that they likely have fewer severe defects that could cause failure. A number of studies calculate ${\bar{\sigma }}_{\left(L_{c}\right)}$ from the Weibull distribution parameters obtained by tensile tests conducted on several filaments at one gauge length [1,2,6,24,26,28], whereas other studies suggest the use of three or four gauge lengths for extrapolation of ${\bar{\sigma }}_{\left(L_{c}\right)}$ [25,28]. Here, we investigated both methods in the determination of the average fibre strength at critical length, ${\bar{\sigma }}_{\left(L_{c}\right)}$.

#### Method 1: Obtaining Weibull Parameters from Fibres at a Single Gauge Length $L_{0}$

The two-parameter Weibull distribution, combined with the weakest link model, is used to present the strength data of the E-glass fibres used in this study:

(A1) $$P=1-\exp\left[-\left(\frac{L}{L_{0}}\right){\left(\frac{\sigma }{\sigma_{0}}\right)}^{m}\right]$$

where P is the probability of failure at the applied tensile strength σ, L is the fibre gauge length and $L_{0}$ is the reference length. The Weibull modulus m, also known as the shape parameter, is a measure of the scatter in the tensile data (higher m means narrower dispersion) and is dimensionless, and $\sigma_{0}$ is a constant known as the scale parameter or characteristic strength [6,26].

For n filaments tested at gauge length $L_{0}$, the strengths of the filaments were ordered from least to the greatest strength, and each data point assigned a rank i. P was determined for each of the n data points by:

(A2) $$P=\frac{i-0.5}{n}$$

For the particular reference gauge length $L_{0}$ used in the test, Equation (A1) can be rearranged to the following:

(A3) $$\ln[-\ln\left(1-P\right)]=m\ln\left(\sigma \right)-m\;\ln\left(\sigma_{0}\right)$$

The Weibull parameters m and $\sigma_{0}$ were determined by plotting ln[−ln(1−P)] vs. ln(σ). The slope of the plot yielded the shape parameter, m, and the scale parameter, $\sigma_{0}$, was calculated from the y-intercept and m. Using the distribution mean, ${\bar{\sigma }}_{L}=\sigma_{0}{\left(\frac{L}{L_{0}}\right)}^{-1/m}\Gamma \left(1+\frac{1}{m}\right)$, where Γ is the gamma function, the average fibre strength ${\bar{\sigma }}_{\left(L_{c}\right)}$ at critical length $L_{c}$ was determined:

(A4) $${\bar{\sigma }}_{\left(L_{c}\right)}={\bar{\sigma }}_{L_{0}}{\left(\frac{L_{0}}{L_{c}}\right)}^{\frac{1}{m}}$$

where ${\bar{\sigma }}_{L_{0}}$ is the average fibre strength at gauge length $L_{0}$.

#### Method 2: Obtaining Weibull Parameters from Filaments at Three Different Gauge Lengths

In order to minimise errors in calculating ${\bar{\sigma }}_{\left(L_{c}\right)}$ from Equation (A4), tensile tests were carried out at three different gauge lengths [28]. Then, the data for $\ln({\bar{\sigma }}_{L})$ vs. ln(L) was plotted, where ${\bar{\sigma }}_{L}$ is the average strength at gauge length L, given by the distribution mean. The mean Weibull shape parameter, m, was calculated as $\left(-\frac{1}{slope}\right)$ from the linear regression obtained from this graph. The scale parameter, $\sigma_{0}$, was obtained by inverting the mean function

(A5) $$\sigma_{0}=\frac{e^{y-intercept}}{\Gamma \left(1+\frac{1}{m}\right)L_{0}{}^{1/m}}$$

The average fibre strength at critical length ${\bar{\sigma }}_{\left(L_{c}\right)}$ could then be calculated by the mean [26,29].

(A6) $${\bar{\sigma }}_{\left(L_{c}\right)}=\sigma_{0}{\left(\frac{L_{c}}{L_{0}}\right)}^{-1/m}\Gamma \left(1+\frac{1}{m}\right)=e^{y-intercept}L_{c}^{-\frac{1}{m}}$$

### Single Fibre Tensile Tests and Results

From previous work in [8], the tensile properties of standalone bead-less glass fibres did not significantly differ from those of beaded fibres. Moreover, since the beads on the fibre are of the same material as the matrix, once the beaded fibre is embedded in the matrix, the strength of fragments of the beaded fibre can be thought of as the same as that of bead-less fibre fragments embedded in an epoxy matrix. Therefore, only the strength of bead-less fibres in air was considered and used going forward.

Single fibre tensile tests were performed on bead-less glass fibres in air on an Instron (model 5965) at a rate of 1 µm s^−1^. Only fibres of diameter 16.8 ± 0.5 µm were used, since this was the chosen fibre diameter for the single fibre fragmentation tests. Glass fibres were stretched taut and glued to a plastic tab using a stiff cyanoacrylate (CN, Tokyo Measuring Instruments laboratory, Tokyo, Japan), as seen in Figure A1a. Double-sided tape was then placed over the tab and the fibre (Figure A1b) before the sample was placed within the clamps of the Instron to prevent the tab from slipping through the clamps. One side of the tab was cut before loading onto the Instron for ease of handling. Once both sides of the fibre were placed in the clamps, the tab was cut on the other side as well so that the fibre was free-standing (Figure A1c). The fibre was then stretched until failure occurred, and the maximum tensile stress at failure (i.e., tensile strength) was recorded.

*Method 1:* An initial gauge length of $L_{0}$ = 30 mm was chosen for this method and 29 filaments were tested at this gauge length (i.e., n = 29). A plot of ln[−ln(1−P)] vs. ln(σ) is given in Figure A2a. The shape parameter, m, the scale parameter, $\sigma_{0}$, and the average fibre strength at critical length for the fibres, ${\bar{\sigma }}_{\left(L_{c}\right)}$ were determined and recorded in Table A1. The average fibre strength at $L_{0}$ = 30 mm was found to be 1344 ± 283 MPa.

*Method 2:* Filaments at two additional gauge lengths 20 mm and 10 mm were tested, with 21 and 25 filaments, respectively, and a plot of $\ln\left({\bar{\sigma }}_{L}\right)$ vs. ln(L) is seen in Figure A2b. The shape parameter, m, the scale parameter, $\sigma_{0}$, and the average fibre strength at critical length for the fibres, ${\bar{\sigma }}_{\left(L_{c}\right)}$, can be found in Table A1.

**Table A1:** Weibull parameters of bead-less E-glass fibres obtained from Methods 1 and 2.

		Method 1	Method 2
Scale parameter (m)		4.69	5.10
Shape parameter ($\sigma_{0}$) (MPa)		1466	3007
${\bar{\sigma }}_{\left(L_{c}\right)}$ Control (MPa)	$L_{c}$ = 0.52 mm	3179 ± 86	3131 ± 79
${\bar{\sigma }}_{\left(L_{c}\right)}$ Beaded fibre (MPa)	$L_{c}$ = 0.45 mm	3272 ± 74	3217 ± 68

Both methods of determining ${\bar{\sigma }}_{\left(L_{c}\right)}$ for fibres for this epoxy-fibre systems appear to agree very well with each other, and therefore, both may be used as a means of determining ${\bar{\sigma }}_{\left(L_{c}\right)}$ of a fibre. A *t*-test comparing the values of the ${\bar{\sigma }}_{\left(L_{c}\right)}$ calculated by each of the methods revealed a *p*-value of 0.048, implying that there is a significant difference between them. Thus, for the purpose of consistency, only the values of ${\bar{\sigma }}_{\left(L_{c}\right)}$ from method 1 were taken in the calculation of τ in Section 3.2.

Similarly, ${\bar{\sigma }}_{\left(L_{c}\right)}$ of beaded fibres was calculated using both methods, as seen in Table A1, which is essentially calculating the strength of the glass fibre at a lower $L_{c}$. This is based on our assertion that the fibre strength of a beaded fibre embedded in a matrix should not be affected by the presence of the beads at the surface. Comparing the ${\bar{\sigma }}_{\left(L_{c}\right)}$ of beaded fibres obtained from methods 1 and 2 using the *t*-test revealed a *p*-value of 0.009, again implying that there is a significant difference between the values obtained from method 1 and method 2. Therefore, we used the results of method 1, as for the beadless fibres.

## Appendix B

During the fabrication process of the specimens, residual stresses and strains are introduced in the fibre and matrix due to the mismatch in thermal coefficients of expansion of the matrix and glass fibres. A typical value of the coefficient of thermal expansion (CTE) of a glass fibre used in this study is $5\times 10^{-6}\;{}^{\circ}C^{-1}$ [32]. The epoxy system used in this study had a glass transition temperature ($T_{g}$) of about 78 °C [8] and was cured at 100 °C, i.e., above $T_{g}$. It is known at the CTE of polymers above $T_{g}$ is much higher than below it due to the polymers being in a more rubbery state above $T_{g}$ [34,35]. Typical CTE values of epoxy are $60\times 10^{-6}\;{}^{\circ}C^{-1}$ below $T_{g}$ and $180\times 10^{-6}\;{}^{\circ}C^{-1}$ above $T_{g}$ [36]. An assumption was made that there was no relaxation of thermal stresses even above $T_{g}$, and so Equation (A7) (modified from [33]) was used to calculate the thermal residual strain on cooling the sample to room temperature:

(A7) $$\varepsilon_{th,f}=\left(\alpha_{m1}-\alpha_{f}\right)\left(T_{g}-T_{cure}\right)+\left(\alpha_{m2}-\alpha_{f}\right)\left(T-T_{g}\right)$$

where $\alpha_{m1}$ and $\alpha_{m2}$ are the CTE of the matrix above and below $T_{g}$, respectively, $\alpha_{f}$ is the CTE of the fibre, T is room temperature (25 °C) and $T_{cure}$ is the temperature at which the sample was cured. $\varepsilon_{th,f}$ was found to be −0.67%.

To calculate the percentage strain ($\varepsilon_{w}$) induced in the fibres by hanging 10 g weights on either end when making the samples for the single fibre fragmentation tests as described in Section 2.3.2, Equation (A8) was used

(A8) $$\varepsilon_{w}=\frac{F}{A\times E}$$

where F is the force induced in the fibre, 10 × 10^−3^ × 9.8 N, A is the area of the fibre of diameter 16.8 µm, and E, the Young’s modulus of the fibre, which from the single fibre tests at gauge length 30 mm done in Appendix A was found to be 68.2 GPa. The $\varepsilon_{w}$ induced in the fibre by the 10 g weights was calculated to be 0.65%, which effectively counterbalances the residual strain induced due to thermal stresses. Therefore, pre-straining the fibres with 10 g weights is important in order to offset the thermal residual strain induced during sample preparation.

### Compressive Residual Strain in Fibre due to Beads

We ask the question of whether or not the beads also contribute to the residual strain in the fibre, especially since they are cured once on the fibre and then cured a second time during sample preparation (see Section 2.3.2). We observed in our previous study [8] that epoxy cured twice was found to have a slightly higher $T_{g}$ of 80 °C (2 °C above the matrix value). We also estimate that the values of $\alpha_{m1}$ and $\alpha_{m2}$ for the beads are the same as for the matrix, because a small difference in $T_{g}$ is negligibly associated with the curing degree at high levels of curing [39]. Using this $T_{g}$, the compressive strain induced by the bead on the fibre would be −0.65% (Equation (A7)), which is a difference of only 0.02% from that of bulk epoxy cured once, equivalent to about 0.43% of the fibre strength, a negligible stress level. Furthermore, this value is highly exaggerated because the bead is small compared to the bulk matrix, and so most of the difference in thermal expansion will be borne by the bead and not by the fibre.

## Appendix C

We calculated in our previous paper [8], based on pullout tests of fibres with single beads, that the friction interfacial stress at the bead front half is $\tau_{f}=64.8\;\mathrm{MPa}$, and that the ratio between the friction interfacial stress ($\tau_{f}$) and the bonding interfacial strength ($\tau_{i}$) is $\tau_{f}/\tau_{i}≅1.24$. Thus, in a fibre with several beads, the average interfacial strength can be calculated by weighing the stress components by their respective action lengths, so that $\tau_{i}$ acts along λ, the distance between bead centres (i.e., the beads period), whereas $\tau_{f}$ acts along L, the bead half-length.

(A9) $$\tau_{beaded}=\frac{\tau_{i}\lambda +\tau_{f}L}{\lambda }=\tau_{i}+\frac{L}{\lambda }\tau_{f}\;$$

The equation can be normalised by $\tau_{i}$:

(A10) $$\frac{\tau_{beaded}}{\tau_{i}}=1+\frac{L}{\lambda }\frac{\tau_{f}}{\tau_{i}}$$

From this study, an average value for 2L was calculated to be 65 µm, and average λ was taken to be 125 µm. Thus, the ratio of half-length to bead period, L/λ≅ 0.26. Using the ratio of $\tau_{f}/\tau_{i}$ from above, we calculate the ratio between $\tau_{beaded}$ and the control as:

(A11) $$\frac{\tau_{beaded}}{\tau_{i}}=1+\left(0.26\times 1.24\right)=1.32$$

Thus, this calculation predicts that the effective shear strength will be higher by about 30% in the beaded fibres compared to the bead-less ones. This prediction, however, is not realised in fibre fragments with more than two beads, because the outer beads carry most of the load whereas the inner beads bear much less (see discussion in the main text, Section 4.1).
